# Supplementary material for: Clonal Hematopoiesis Mutations Are Present in Atherosclerotic Lesions in Peripheral Artery Disease
Source: Int J Mol Sci. 2023 Feb 16;24(4):3962. doi: 10.3390/ijms24043962 (PMC9963103; doi:10.3390/ijms24043962)

Clonal Hematopoiesis Mutations Are Present in Atherosclerotic Lesions in Peripheral Artery Disease

Petra Büttner [1,*], Julia Böttner [1], Knut Krohn [2], Ronny Baber [3,4], Uwe Platzbecker [5], Michael Cross [5], Steffen Desch [1], Holger Thiele [1], Sabine Steiner [6,7], Dierk Scheinert [6], Klaus H. Metzeler [5] and Daniela Branzan [7,8]

1 Department of Cardiology, Heart Center Leipzig at University of Leipzig, University of Leipzig, Strümpellstr. 39, 04289 Leipzig, Germany

2 Core Unit DNA-Technologies, Medical Faculty, University of Leipzig, Philipp-Rosenthal-Str. 55, 04103 Leipzig, Germany

3 Institute of Laboratory Medicine, Clinical Chemistry and Molecular Diagnostics, University of Leipzig, Paul-List-Strassse 13-15, 04103 Leipzig, Germany

4 Leipzig Medical Biobank, University of Leipzig, Strümpellstr. 39, 04289 Leipzig, Germany

5 Department of Hematology, Cellular Therapy and Hemostaseology, Leipzig University Hospital, Liebigstrasse 20, 04103 Leipzig, Germany

6 Division of Angiology, Department of Internal Medicine, Neurology and Dermatology, Leipzig University Hospital, Liebigstrasse 20, 04103 Leipzig, Germany

7 Helmholtz Institute for Metabolic, Obesity and Vascular Research (HI-MAG) of the Helmholtz Zentrum Munich at the University of Leipzig and University Hospital Leipzig, Rosenthal-Straße 27, 04103 Leipzig, Germany

8 Visceral, Transplantation, Thorax and Vascular Surgery, Leipzig University Hospital, Liebigstrasse 20, 04103 Leipzig, Germany

* Correspondence: petra.buettner@medizin.uni-leipzig.de

Supplementary Methods

A total of 289 samples were analyzed for CH mutations. Initially, variants were filtered by automated calling algorithms. If a variant was detected in at least one sample, all samples were manually analyzed for this variant. The variant chr4_105235051 was detected exclusively in samples from patient 1. All other variants were also detected in samples from other patients with very low VAFs, likely representing background noise in sequencing. The characteristics of background noise, i.e., the number of samples where the variant was detected and the allele frequency of these variants in samples that were not from the index patient, are highly variable (Figure S1). Most variants were detected in many samples with low VAF. True positive variant calls are characterized by higher VAF, and clustering in samples from one patient. Therefore, for each variant, specific detection limits were determined. Means and confidence intervals of the allele frequency of each variant across all samples were calculated, and the upper boundary of the 99.9% confidence interval was used to define variant-specific cut offs (Table S1). This approach was appropriate for eight variants to exclude false positive and identify true positive samples (see Figure S1). Interestingly, this approach identified variant chr2_25234323 in peripheral blood, the $CD14^+$ fraction and the residual blood cell populations of patient 19, although the variant was initially detected in patient 25. Due to our original screening approach, we did not include these data in the main results. For ten variants, the approach resulted in putative false positive identification of variants in samples from other patients, while no patient-specific patterns were apparent (see Figure S2). For these variants, we applied a fixed VAF cutoff of 1%.

Table S1: Descriptive data for variants detected in this study. The upper limit of the 99.9 confidence interval (CI) of the mean was determined to develop assay specific cut-offs. This approach was appropriate for eight variants (bold). NN – number of samples in which no variant allele was detected, total number of samples = 289.

Variant	Mean VAF	STD VAF	Upper limit of 99.9% CI	Maximum VAF	NN
chr4_105235051	0.00019	0.00191	0.00057	0.03100	257
**chr4_105237044**	**0.00213**	**0.01291**	**0.00467**	**0.15700**	**124**
**chr4_105259638**	**0.00191**	**0.01453**	**0.00476**	**0.19900**	**229**
**chr2_25234307**	**0.00248**	**0.01437**	**0.00531**	**0.14500**	**21**
chr2_25240379	0.00138	0.00682	0.00272	0.06500	68
chr2_25240699	0.00079	0.00437	0.00165	0.04400	160
chr4_105259678	0.00059	0.00752	0.00207	0.12600	258
**chr2_25246671**	**0.00062**	**0.00308**	**0.00123**	**0.03200**	**59**
chr20_32434825	0.00014	0.00126	0.00039	0.01700	276
chr4_105275568	0.00049	0.00356	0.00119	0.06000	80
chr2_25244175	0.00107	0.00180	0.00142	0.02700	9
chr4_105236178	0.00170	0.00500	0.00268	0.05300	34
**chr4_105275086**	**0.00095**	**0.00507**	**0.00195**	**0.05000**	**114**
**chr4_105235270**	**0.00025**	**0.00184**	**0.00061**	**0.02200**	**275**
**chr4_105243673**	**0.00461**	**0.02868**	**0.01025**	**0.26500**	**80**
chr4_105237312	0.00062	0.00390	0.00139	0.05300	232
**chr2_25234323**	**0.00048**	**0.00257**	**0.00098**	**0.04200**	**15**
chr4_105235102	0.00040	0.00158	0.00071	0.01900	85
chr2_25246755	0.00045	0.00204	0.00086	0.02700	98

Table S2: Overview of detected variants in peripheral blood (PB), blood subpopulations, and tissues (SF=subcutaneous fat, PVT=peri-vascular tissue). Columns show: the index patient (IP), the gene, the base position of the mutation, the reference (REF) and the alternative (ALT) variant, the variant consequence, the resulting amino acid (AA) exchange, the number of detected (ALT) and the number of sequenced variants (depth=dep), and the allele frequency (AF) calculated from ALT/dep.

						Blood								Tissue							
						PB		Monocytes		Progenitors		Residual		Plaque		Collateral		SF		PVT	
IP	gene	base position	REF/ALT	consequence of mutation	AA exchange	ALT/dep	AF	ALT/dep	AF	ALT/dep	AF	ALT/dep	AF	ALT/dep	AF	ALT/dep	AF	ALT/dep	AF	ALT/dep	AF
**1**	TET2	105237044	G/GT	frameshift insertion	NM_001127208:exon3:c.3103dupT:p.Q1034fs	20/814	0.0246	204/2110	0.0967	1832/11653	0.1572	75/1345	0.0558	137/6703	0.0204	73/12678	0.0058	241/12537	0.0192	163/8676	0.0188
	TET2	105235051	ATT/A	frameshift deletion	NM_001127208:exon3:c.1110_1111del:p.Y370fs	0/734	0.0000	23/3123	0.0074	0/10072	0.0000	35/1131	0.0309	0/5762	0.0000	0/3305	0.0000	27/11832	0.0023	0/8476	0.0000
**2**	TET2	105259638	G/T	nonsynonymous SNV	NM_001127208:exon7:c.G3823T:p.G1275W	36/912	0.0395	406/2055	0.1976	27/567	0.0476	273/2109	0.1294	0/2355	0.0000	16/1192	0.0134	44/2057	0.0214	69/3143	0.0220
**3**	DNMT3A	25234307	G/T	nonsynonymous SNV	NM_001320893:exon18:c.C2255A:p.P752Q	1146/27577	0.0416	3969/27330	0.1452	173/16526	0.0105	1169/11589	0.1009	1213/25044	0.0484	463/50436	0.0092	544/42272	0.0129	589/38486	0.0153
**4**	DNMT3A	25240379	G/A	nonsynonymous SNV	NM_001320893:exon14:c.C1789T:p.R597C	1493/22854	0.0653	1019/15991	0.0637	l.c.	l.c.	0/11602	0.0000	89/6833	0.0130	51/4820	0.0106	613/20089	0.0305	409/11698	0.0350
**7**	DNMT3A	25240699	A/G	nonsynonymous SNV	NM_001320893:exon13:c.T1658C:p.I553T	435/15120	0.0288	na	na	na	na	na	na	46/3714	0.0124	19/5984	0.0032	66/24287	0.0027	87/23421	0.0037
**8**	DNMT3A	25246671	C/T	nonsynonymous SNV	NM_001320893:exon5:c.G772A:p.A258T	847/31723	0.0267	580/22778	0.0255	0/4329	0.0000	1113/34583	0.0322	46/8745	0.0053	142/15763	0.0090	256/26545	0.0096	182/22268	0.0082
	TET2	105259678	G/A	nonsynonymous SNV	NM_001127208:exon7:c.G3863A:p.G1288D	6/1850	0.0032	84/5037	0.0167	59/468	0.1261	2/1262	0.0016	0/802	0.0000	0/483	0.0000	2/5971	0.0003	6/5770	0.0010
**9**	ASXL1	32434825	GA/G	frameshift deletion	NM_015338:exon12:c.2114delA:p.E705fs	174/10325	0.0169	l.c.	l.c.	l.c.	l.c.	l.c.	l.c.	4/331	0.0121	15/3393	0.0044	0/2112	0.0000	2/700	0.0029
	TET2	105275568	C/A	nonsynonymous SNV	NM_001127208:exon11:c.C5058A:p.S1686R	4/11361	0.0004	3/6373	0.0005	3469/57564	0.0603	0/55	0.0000	8/8265	0.0010	2/4259	0.0005	0/6930	0.0000	2/10615	0.0002
**10**	DNMT3A	25244175	T/C	nonsynonymous SNV	NM_001320893:exon10:c.A1375G:p.N459D	918/34574	0.0266	na	na	na	na	na	na	1072/164897	0.0065	95/21264	0.0045	98/36000	0.0027	390/31468	0.0124
	TET2	105236178	C/T	stopgain	NM_001127208:exon3:c.C2236T:p.Q746X	163/3092	0.0527	na	na	na	na	na	na	350/8864	0.0395	35/1609	0.0218	121/9374	0.0129	319/7829	0.0407
	TET2	105275086	C/T	stopgain	NM_001127208:exon11:c.C4576T:p.Q1526X	207/5876	0.0352	na	na	na	na	na	na	1373/27284	0.0503	194/4389	0.0442	148/12429	0.0119	252/8382	0.0301
**15**	TET2	105235270	CA/C	frameshift deletion	NM_001127208:exon3:c.1329delA:p.T443fs	43/3576	0.0120	266/12010	0.0221	386/25218	0.0153	10/1959	0.0051	32/7890	0.0041	5/1750	0.0029	10/1264	0.0079	2/1028	0.0019
**19**	TET2	105243673	G/A	stopgain	NM_001127208:exon6:c.G3698A:p.W1233X	334/1652	0.2022	13137/49607	0.2648	11581/44954	0.2576	12035/61007	0.1973	114/1959	0.0582	223/2434	0.0916	1737/30081	0.0577	1201/25842	0.0465
**21**	TET2	105237312	G/A	nonsynonymous SNV	NM_001127208:exon3:c.G3370A:p.V1124I	64/2534	0.0253	38/2390	0.0159	23/1368	0.0526	23/1368	0.0168	84/4524	0.0186	6/4197	0.0014	47/5425	0.0087	4/1988	0.0020
**25**	DNMT3A	25234323	G/T	nonsynonymous SNV	NM_001320893:exon18:c.C2239A:p.R747S	602/14248	0.0423	na	na	na	na	na	na	119/32292	0.0037	274/64454	0.0043	84/13389	0.0063	102/21161	0.0048
**26**	TET2	105235103	TC/T	frameshift deletion	NM_001127208:exon3:c.1161delC:p.F387fs	55/2863	0.0192	na	na	na	na	na	na	95/5431	0.0175	25/8158	0.0031	26/5494	0.0047	12/6480	0.0019
**28**	DNMT3A	25240699	A/G	nonsynonymous SNV	NM_001320893:exon13:c.T1658C:p.I553T	418/12224	0.0342	66/3643	0.0181	3/113	0.0265	128/6293	0.0203	692/15806	0.0438	4/1519	0.0026	58/15047	0.0039	50/17080	0.0029
	DNMT3A	25246755	T/A	stopgain	NM_001320893:exon5:c.A688T:p.K230X	150/11325	0.0132	48/5659	0.0085	6/746	0.0080	46/3959	0.0116	138/5086	0.0271	1/15469	0.0001	15/15644	0.0010	49/10401	0,0047

Figure S1: Distribution of allele frequencies (y-axis) of eight variants initially detected in at least one sample from one patient in all samples that were included in this study. Dashed lines indicate the upper limit of 99.9 confidence interval. White circles indicate samples of the index patient. Black circles indicate samples from other patients. Squares indicate three samples from patient 19 found to harbor the same variant as the index patient 25.

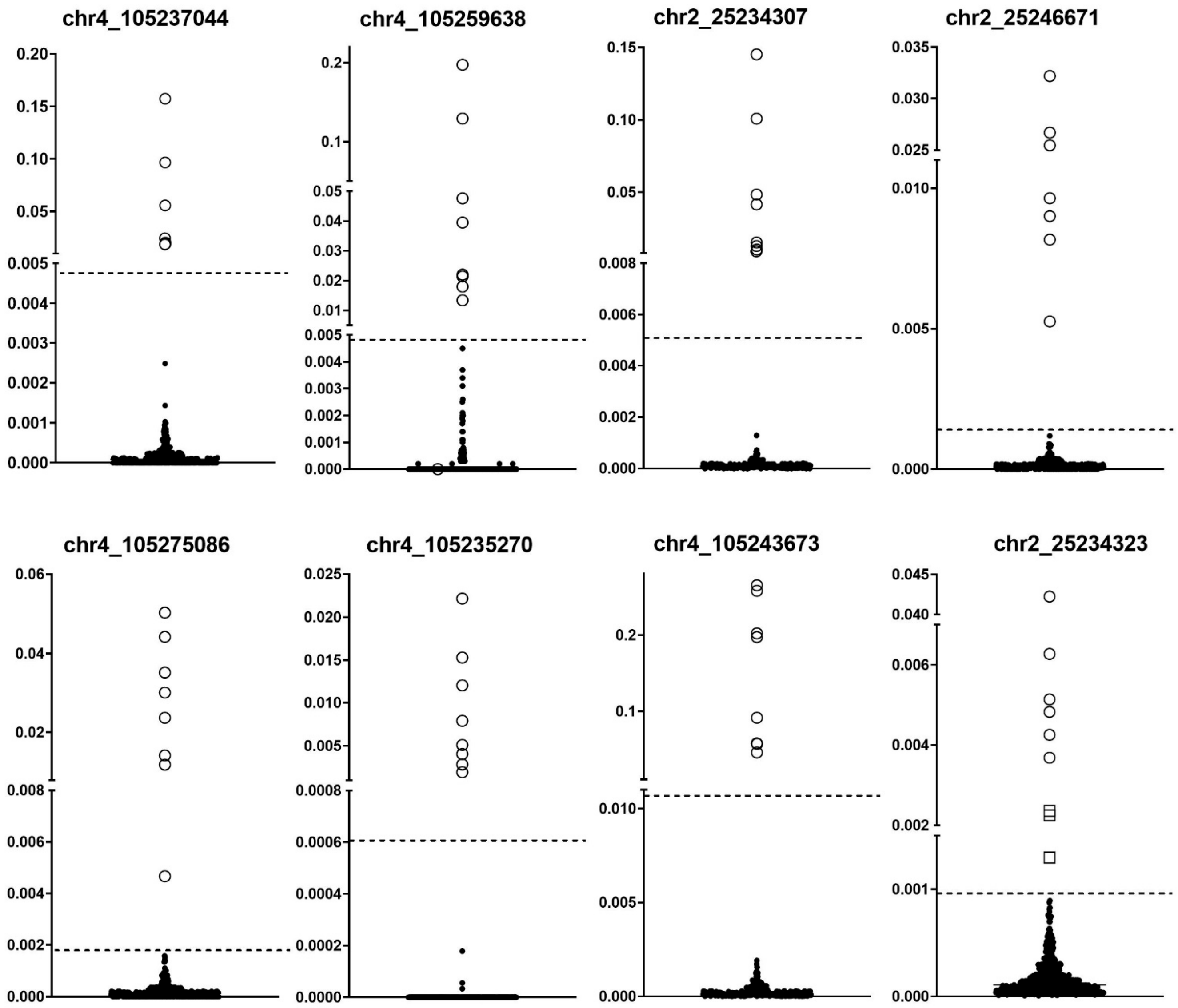

Figure S2: Distribution of allele frequencies (y-axis) of ten variants in all samples that were included in this study. Dashed lines indicate the upper limit of 99.9 confidence interval; solid line indicates an AF of 1%. White circles indicate samples of the index patient. Black circles indicate samples from other patients. The Variant chr2_25240699 (right side) was found in patient 7 (white squares) and 28 (white circles).

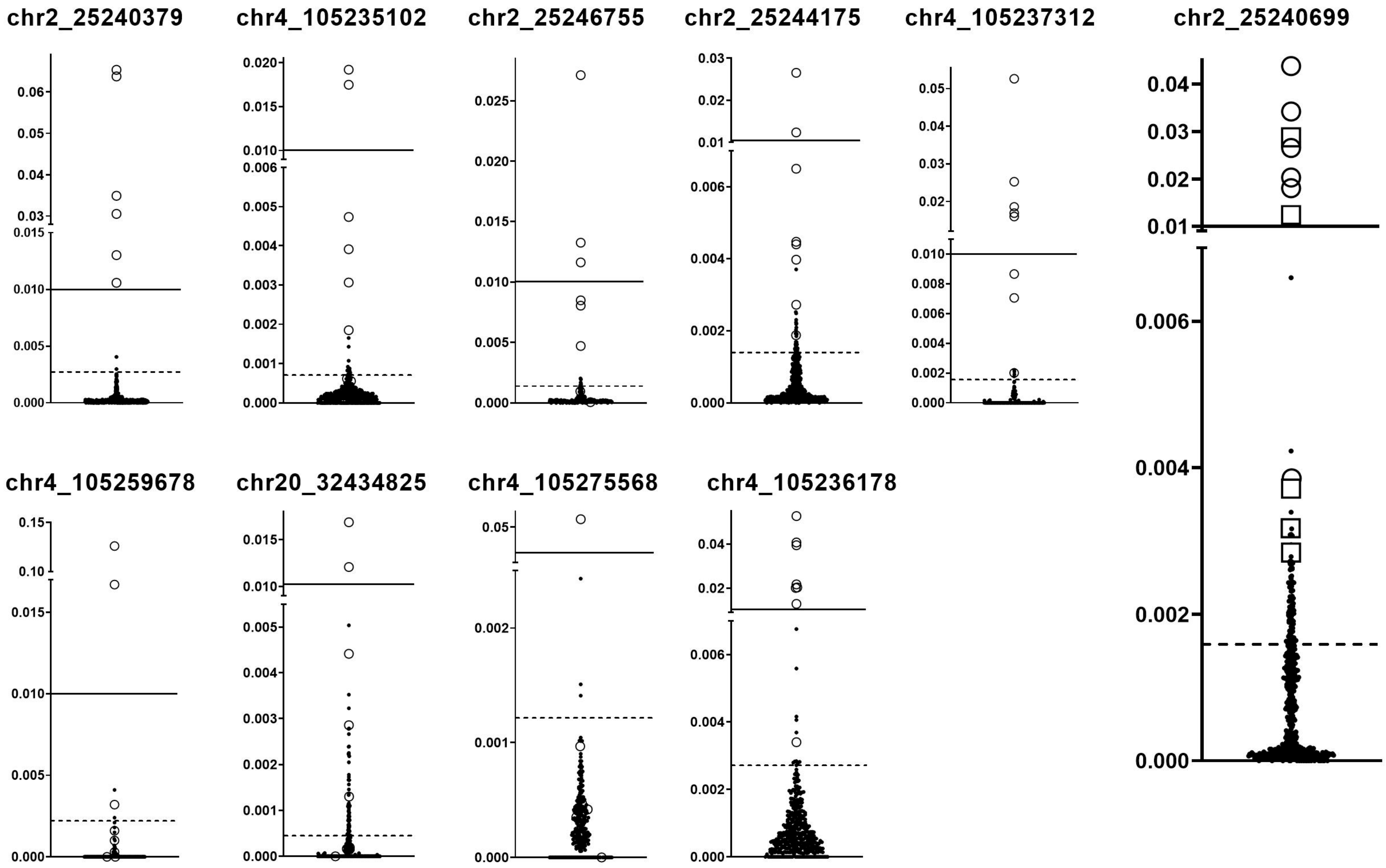

Supplement: Supplementary file 1 [file ijms-24-03962-s001.zip › ijms-2200640-supplementary.pdf]
